# 8-, 9-, and 11-Aryloxy Dimeric Aporphines and Their Pharmacological Activities

**DOI:** 10.3390/molecules26154521

**Published:** 2021-07-27

**Authors:** Ghada Ali, Gregory D. Cuny

**Affiliations:** 1 Department of Chemistry, College of Natural Sciences and Mathematics, University of Houston, Houston, TX 77204, USA; gali2@uh.edu; 2 Department of Pharmacological and Pharmaceutical Sciences, College of Pharmacy, University of Houston, Houston, TX 77204, USA

**Keywords:** dimeric aporphines, aryloxy, cytotoxicity, pharmacological activity

## Abstract

Aporphines, a major group of aporphinoid alkaloids, exhibit interesting and diverse pharmacological activities. A set of dimeric aporphines with an aryloxy group at C8, C9, and C11 have been isolated from six genera and shown to elicit various biological activities such as antitumor, antimalarial, antimicrobial, antiplatelet aggregation, antifibrotic, immunosuppressive, and vasorelaxant properties. In this review, the nomenclature, chemical structures, botanical sources, pharmacological activities, and synthetic approaches of this set of dimeric alkaloids are presented.

## 1. Introduction

Aporphinoid alkaloids represent one of the largest subclasses of benzylisoquinoline alkaloids, with more than 600 members isolated [1,2,3,4,5]. They are widely distributed among more than 25 families of flowering plants, trees, and shrubs including Annonaceae, Araceae, Aristolochiaceae, Berberidaceae, Canellaceae, Euphorbiaceae, Eupomatiaceae, Fumariaceae, Hernandiaceae, Hypecoaceae, Lauraceae, Leguminosae, Liliaceae, Magnoliaceae, Menispermaceae, Monimiaceae, Nelumbonaceae, Nymphaeaceae, Papaveraceae, Piperaceae, Ranunculaceae, Rhamnaceae, Rutaceae, Saururaceae, Sabiaceae, Siparunaceae, and Symplocaceae [6,7]. Aporphinoids can be categorized into several classes according to their chemical structures. These subclasses include aporphines, dimeric aporphines, proaporphines, oxoaporphines, 4,5-dioxoaporphines, oxoisoaporphines, phenanthrenes, aristolochic acids, aristolactams, and miscellaneous aporphines [6].

**Figure 1:**
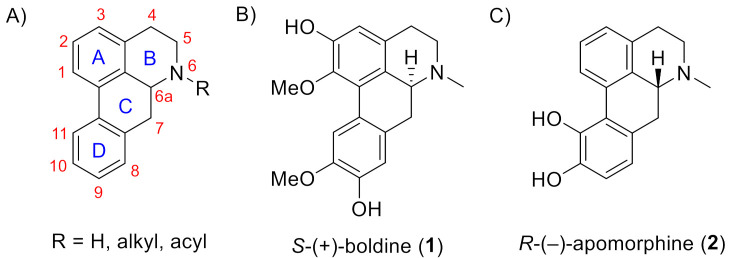
(**A**) Tetracyclic skeleton of aporphines with IUPAC numbering. (**B**) Structure of *S*-(+)-boldine (**1**). (**C**) Structure of *R*-(−)-apomorphine (**2**).

Aporphines are the main class of aporphinoid alkaloids. Their basic framework, as shown in Figure 1A, consists of four fused-ring systems with rings A, B, and D constituting an embedded 1-benzyltetrahydroisoquinoline. They can be further classified into four subgroups according to their chemical structure: aporphines sensu stricto, 6a,7-dehydroaporphines, 7-alkyl aporphines, and 7-oxygenated and/or 4-oxygenated aporphines [6]. Naturally occurring and synthetic aporphines possess a wide range of biological activities such as anticancer, anti-inflammatory, antioxidant, antiplatelet, anti-arrhythmic, antiprotozoal, antiplasmodial, antiviral, antibacterial, antimalarial, antiparkinsonian, and anticonvulsant properties [6]. Over the last few decades, several reviews covering the pharmacological activities of aporphines such as their antioxidant [8], cytotoxic [9,10,11,12], antiviral, antibacterial, and antifungal properties [13] as well as their dopaminergic, noradrenergic, and serotonergic activities [14,15,16] have been published. Additionally, the potent free radical scavenger properties of *S*-(+)-boldine (**1**) (Figure 1) and its numerous pharmacological activities have been reviewed [17,18]. Moreover, aporphine alkaloids have served as leads for the development of potential pharmaceutical products. For instance, the synthetic aporphine, *R*-(−)-apomorphine (**2**), shown in Figure 1, is a potent dopamine D_1_ and D_2_ receptor agonist that stimulates locomotor behavioral activity. This potent activity supported its approval as a drug (Apokyn^®^) for the treatment of advanced Parkinson’s disease [19,20,21]. Recently, *R*-(−)-apomorphine was also recommended as a potential treatment for Alzheimer’s disease [19,22].

Dimeric aporphines are another class of aporphinoid in which two aporphinoid units are usually connected by either an ether or less frequently a carbon–carbon bond. According to the type and location of this bond, they can be divided into several subgroups [6,23,24,25]:

(1) Carbon–carbon bonded dimers, where the two aporphinoid units are coupled by a C–C bond. They include:
  i. Bisaporphines: the two aporphine units form either 7,7′-, 7,4′-, or 8,8′- linked dimers.
  ii. Aporphine–benzylisoquinoline dimers (uskudaramine type): these compounds have a C–C bond between C8 of the aporphine unit and C11’ of the benzylisoquinoline moiety.
(2) Ether-bonded dimers, where the two halves of the dimer are connected by an ether linkage. They include:
  i. Bisaporphines: the two aporphine units are linked together by an ether bond that may be formed between C1 and C2’, C8 and C9’, or C8 and C11’.
  ii. Proaporphine–benzylisoquinoline dimers: this group includes pakistanamine- and epivaldiberine-type dimers. The ether bond is located between C9 of the proaporphine moiety and C12’ of the benzylisoquinoline unit in the case of pakistanamine-type dimers. However, it is located between C11 of the proaporphine unit and C12’ of the benzylisoquinoline moiety in epivaldiberine-type dimers.
  iii. Aporphine–phenyl (hernandaline-type) and proaporphine–phenyl (coyhaiquine-type) dimers: hernandaline-type dimers have a substituted phenyl ether linked to an aporphine moiety at C9. Additionally, the phenyl group can be connected to the aporphine unit at C1 or C8. On the other hand, coyhaiquine-type dimers possess a substituted phenyl ether connected to C9 of a proaporphine unit. These two rare subgroups are probably formed via the oxidation of the benzylisoquinoline half of the dimer into hydroxyl methyl, aldehyde, carboxylic acid, or ester-substituted phenyl ethers.
  iv. Aporphine–protoberberine dimers: the protoberberine unit is coupled to the aporphine moiety at either C8 or C9.
  v. Aporphine–pavine dimers: the pavine unit is connected to the aporphine moiety at C9.
  vi. Aporphine–benzylisoquinoline dimers: this is the largest subgroup of dimeric aporphines. Based on the nature of the tetrahydroisoquinoline moiety (*S*-reticuline or *S*-coclaurine), from which each monomer can be derived, and the location of the ether linkage between the two halves of the dimer, aporphine–benzylisoquinoline dimeric alkaloids can be divided into: (1) reticuline–reticuline dimers (thalicarpine and fetidine types), (2) reticuline–coclaurine dimers (istanbulamine and thalifaberine types), and (3) coclaurine–coclaurine dimers (pakistanine and kalashine types). Table 1 illustrates the position of the ether bond in each type.

This review presents the nomenclature, chemical structures, botanical sources, pharmacological activities, and synthetic approaches of some types of ether-linked dimers. We focus our discussion on the naturally-occurring dimers in which an aporphine (including 6a,7-dehydroaporphines) unit is linked at C8, C9, or C11 via an ether linkage to either aporphine, pavine, protoberberine, phenyl, or 1-benzyltetrahydroisoquinoline units.

## 2. C8-Aryloxy Aporphines and 6a,7-Dehydroaporphines

### 2.1. Chemical Structure Classification and Occurrence

**Figure 2:**
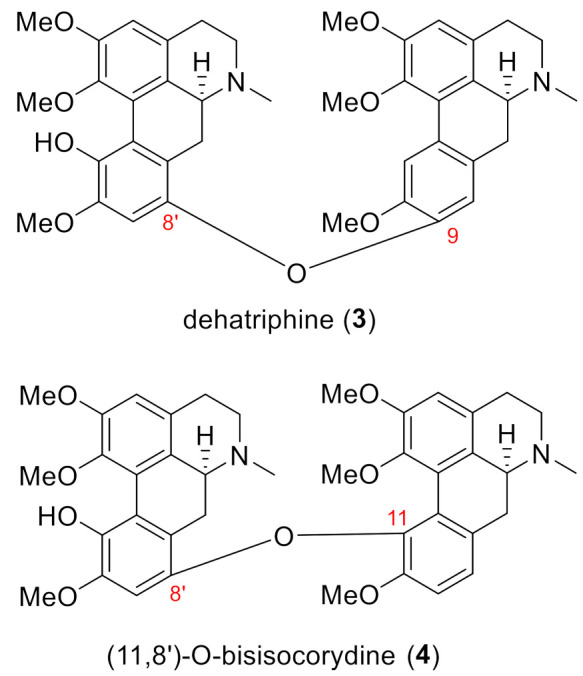
Bisaporphines with ether linkage at the C8 position.

**Figure 3:**
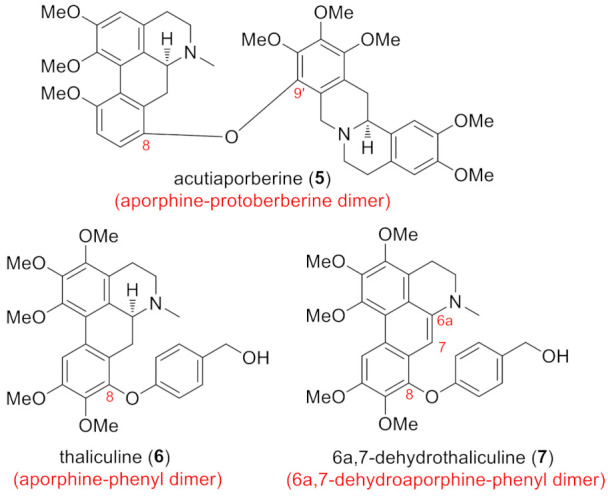
Structures of acutiaporberine (**5**), thaliculine (**6**), and 6a,7-dehydrothaliculine (**7**).

A set of dimeric aporphines and 6a,7-dehydroaporphines with an aryloxy substituent at C8 have been mainly isolated from several species of the *Thalictrum* genus (Ranunculaceae) or *Dehaasia triandra* (Lauraceae). To the best of our knowledge, thirty-two compounds belonging to C8-aryloxy aporphines and six compounds belonging to C8-aryloxy 6a,7-dehydroaporphines have been reported to date. This set of compounds can be classified according to its chemical structure into four groups:

(1) Bisaporphines: only two isolated bisaporphines (Figure 2) with an ether linkage at C8 of one of the aporphine units have been reported. They were isolated in 1996 from the leaves of *Dehaasia triandra*. The first bisaporphine, named dehatriphine (**3**), has an ether linkage at the C8’ position of isocorydine and the C9 position of N-methyl-laurotetanine [26], while the second compound, named (11,8′)-O-bisisocorydine (**4**), has two isocorydine units connected by an ether bond at C8’ and C11 of the two units [27].
(2) Aporphine–protoberberine dimeric alkaloid: acutiaporberine (**5**) is the only known bisalkaloid of this class (Figure 3). It was isolated in 2000 from the root of *Thalictrum acutifolium* (Hand.-Mazz.) Boivin [28].
(3) Aporphine and 6a,7-dehydroaporphine–phenyl dimeric alkaloids: thaliculine (**6**) and 6a,7-dehydrothaliculine (**7**), shown in Figure 3, represent the only reported examples to date with an ether bond at C8 of the aporphine unit. They were isolated together in 2019 from the roots of *Thalictrum cultratum* [29].
(4) Aporphine and 6a,7-dehydroaporphine–benzylisoquinoline dimers: this is the largest group of C8-aryloxy aporphines. To date, thirty-three members belonging to this group have been reported. The names, chemical structures, and botanical sources of these compounds are shown in Table 2, Table 3, Table 4 and Table 5.

The aporphine–benzylisoquinoline dimeric alkaloids **8**–**29** [30,31,32,33,34,35,36,37,38,39,40,41,42] listed in Table 2 belong to the group of thalifaberine-type dimers in which the ether bond is located between C8 of the aporphine unit and C12’ of the benzylisoquinoline moiety. (+)-Thalifaberine (**8**) and (+)-thalifabine (**9**) were the first two thalifaberine-type dimers reported in the literature [30]. This group shares some common structural features: (1) all of them have (6aS, 1′S) absolute configurations; (2) the aporphine moiety has oxygenated substituents (hydroxy or methoxy) at C1, C2, C9, and C10, and many of these compounds also have an oxygenated substituent at C3; and (3) the 1-benzyltetrahydroisoquinoline unit has oxygenated substituents (hydroxy, methoxy, or methylenedioxy) at C6’ and C7’. Additionally, C5’ may have an oxygenated substituent (hydroxy, methoxy, or methylenedioxy).

Table 3 shows four aporphine–benzylisoquinoline dimers **30**–**33** with a 2′-*N*-oxide functional group [42]. In addition, five 6a,7-dehydroaporphine–benzylisoquinoline dimers **34**–**38** [31,40,42] with unsaturation between C6a and C7 are listed in Table 4. All of the compounds listed in Table 3 and Table 4 are thalifaberine-type dimers and have the same common structural features discussed above.

Lee and Doskotch [43] reported the isolation of two new benzylisoquinoline–aporphine dimers **39** and **40** (Table 5), which are structurally distinct from the thalifaberine-type dimers and do not belong to any of the other subtypes of benzylisoquinoline–aporphine dimers listed in Table 1. Both the aporphine and benzylisoquinoline units are reticuline-derived, and the ether linkage is between C11’ of the monosubstituted 12′-benzylisoquinoline moiety and C8 of the aporphine unit, which is also oxygenated at C10 and C11.

### 2.2. Biological Activities of C8-Aryloxy Aporphines and 6a,7-Dehydroaporphines

Nearly all of the isolated aporphine and 6a,7-dehydroaporphine–benzylisoquinoline dimeric alkaloids have exhibited cytotoxicity activity [30,34,35,36,38,39,42]. For example, (+)-thalifaberine (**8**) showed cytotoxicity against Walker-256 carcinoma cells [30]. Additionally, (+)-thalifalandine (**18**) exhibited significant cytotoxicity against P-388 and L-1210 leukemia cells with IC_50_ values of 0.7–1.8 μg/mL [34]. In another study, (+)-thalifarazine (**17**) displayed a range of cytotoxicities against human KB, HCT-8 colon tumor, A-549 lung carcinoma, murine P-388, and L-1210 lymphocytic leukemia cell lines, with ED_50_ values of 1.5, 430, 6.53, 3.75, and 5.69 μg/mL, respectively [36]. Additionally, the cytotoxicity activities of (+)-thalifaberine (**8**), (+)-thalifaberidine (**20**), and (+)-thalifasine (**12**) were evaluated against twelve cell lines (BCA-1, HT-1080, Lu-1, Col-2, KB, KB-V (+VLB), KB-V (-VLB), P-388, A431, LNCaP, ZR-75-1, and U373) and showed significant cytotoxicity activities, with ED_50_ values ranging from 0.6 to 17.7 μg/mL [38]. Moreover, ten dimeric aporphines—**9**–**11**, **13**–**14**, **16**–**19**, and **38**—showed cytotoxicity activity against the P-388 cell line, with ED_50_ values in the range of 1.0–1.5 μg/mL [38]. Another study revealed that thalifaboramine derivatives **22–26** possess cytotoxicity activity against several cell lines (Lu-1, KB, KB-V (+VLB), LNCaP, and ZR-75-1), with ED_50_ values ranging from 0.5 to 11.2 μg/mL [39]. Recently, the antiproliferative activities of (+)-thalifaberine (**8**), (+)-thalifabatine (**11**), (+)-thalifaronine (**13**), and (+)-thalicultratines A–K (**27–37**) were assessed against human prostate cancer PC-3 and leukemia HL-60 cell lines [42]. All the tested compounds showed growth inhibitory activity against these cells, with IC_50_ values in the ranges of 1.01–5.05 and 2.47–18.66 μM, respectively. Additionally, this study showed that 2′-*N*-oxide aporphine–benzylisoquinoline alkaloids **30**–**33** and 6a,7-dehydroaporphine–benzylisoquinoline dimeric alkaloids **34**–**38** are less active than their parent aporphine–benzylisoquinoline alkaloids **8** and **27**–**29**.

The aporphine–protoberberine dimer, acutiaporberine (**5**), exhibited cytotoxicity activity against four human carcinoma cell lines: NSCLC (IC_50_ = 80 μg/mL), MGC (IC_50_ = 65 μg/mL), LCC (IC_50_ = 60 μg/mL), and HeLa (IC_50_ = 37 μg/mL) [44]. In addition, it induced apoptosis in the human metastatic lung cancer cell line 95-D [45] and the human non-small cell lung cancer cell line PLA-801 [46]. Moreover, the aporphine–phenyl dimer thaliculine (**6**) showed weak cytotoxicity activity against the HL-60 cell line, with an IC_50_ value of 31.40 μM compared to 5-fluorouracil (IC_50_ = 6.55 μM) [29].

In addition to cytotoxicity activity, some aporphine–benzylisoquinoline dimers have exhibited antimalarial activity [38,39]. In one study, (+)-thalifaberine (**8**), (+)-thalifaberidine (**20**), and (+)-thalifasine (**12**) showed antimalarial activities against chloroquine-sensitive (D-6) and chloroquine-resistant (W-2) clones of *Plasmodium falciparum*. Out of the three tested compounds, dimeric aporphine **12** showed the most potent antimalarial activity, with ED_50_ (W-2) = 49.3 and (D-6) = 238 ng/mL [38]. In another study, the antimalarial activity of thalifaboramine derivatives **22**–**23** and **26** were evaluated against (D-6) and (W-2) clones of *P. falciparum*. All the tested compounds showed antimalarial activity, with ED_50_ (W-2) values ranging from 10.2 to 24.2 ng/mL and ED_50_ (D-6) ranging from 112 to 176 ng/mL [39].

### 2.3. Reported Synthetic Approaches for C8-Aryloxy Aporphines

**Scheme 1:**
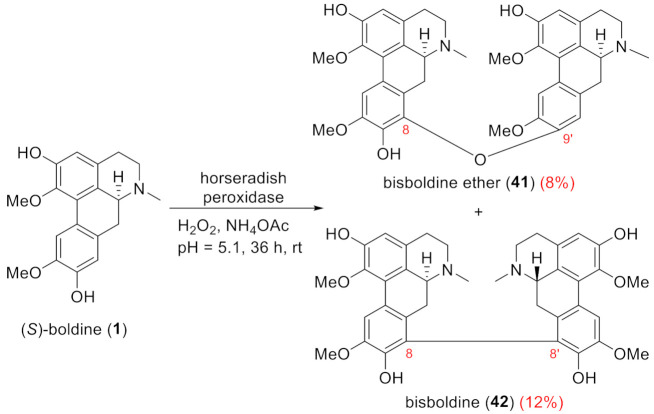
Enzyme-catalyzed synthesis of bisboldine ether (**41**) from *S*-boldine (**1**).

Very few approaches for the synthesis of the 8-aryloxy aporphine scaffold have been reported. The earliest route for the formation of a non-naturally occurring ether-linked bisaporphine was described for the first time by Stuart and Callender in 1972 using enzymatic catalyzed oxidation (Scheme 1). Horseradish peroxidase was used to convert *S*-boldine (**1**) into bisboldine ether (**41**) and bisboldine (**42**) in 8% and 12% yields, respectively [47]. 

**Scheme 2:**
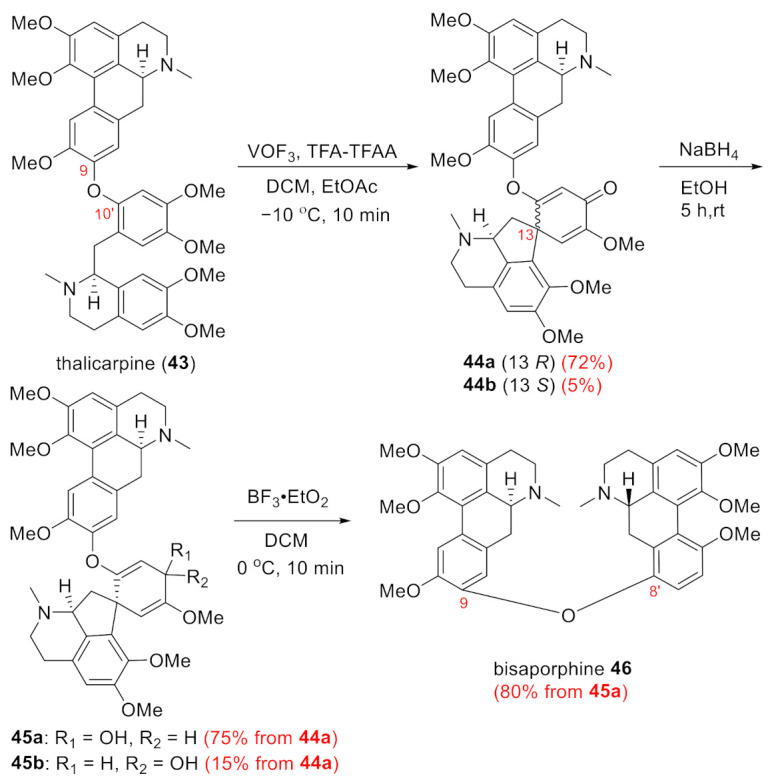
Synthesis of bisaporphine **46** from thalicarpine (**43**). TFA: trifluoroacetic acid; TFAA: trifluoroacetic anhydride; DCM: dichloromethane.

Another method for the synthesis of C8/C9’ ether-linked bisaporphine was reported in 1978 (Scheme 2). Because of the potent activity of thalicarpine (**43**) as an anticancer agent and the need to understand its structure–activity relationship (SAR), Kupchan et al. reported the synthesis of three synthetic analogs; one of them was the bisaporphine **46**. In their synthesis, the non-phenolic oxidative coupling of the tetrahydroisoquinoline unit of thalicarpine (**43**) using vanadium oxytrifluoride (VOF_3_) gave the corresponding aporphine–proaporphine dimers **44a,b**, epimeric at C13, as two separable diastereomers in 72% and 5% yields, respectively. The sodium borohydride reduction of **44a** furnished dienols **45a**,**b** as two separable diastereomers in 75% and 15% yields, respectively. The dienol–benzene rearrangement of **45a** with boron trifluoride etherate at 0 °C for 10 min gave bisaporphine **46** as the corresponding dihydrobromide salt in an 80% yield [48].

**Scheme 3:**
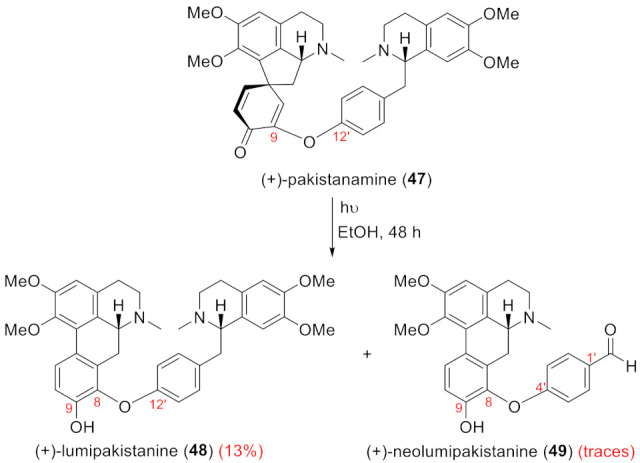
Photolysis of proaporphine–benzylisoquinoline dimeric alkaloid **47**.

In a later study, Hussain et al. described the first photo-catalyzed conversion of a proaporphine–benzylisoquinoline dimer into an aporphine–benzylisoquinoline dimer, as shown in Scheme 3. The sunlight irradiation of an ethanolic solution of (+)-pakistanamine (**47**) for 48 h furnished the non-natural dimer (+)-lumipakistanine (**48**) in a 13% yield, along with a trace amount of (+)-neolumipakistanine (**49**) [49]. 

**Scheme 4:**
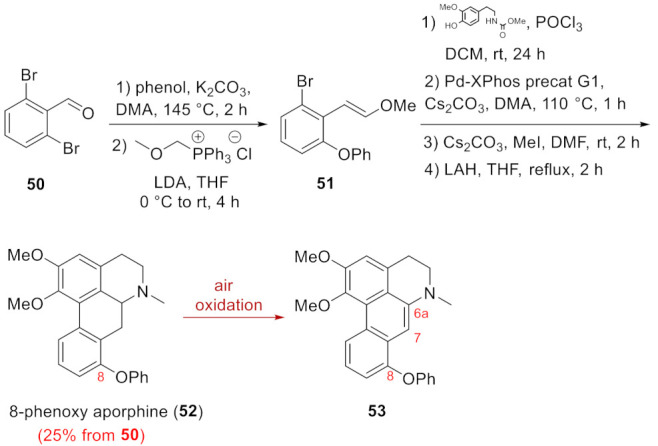
Synthesis of 8-phenoxy aporphine model substrate (**52**). DMA: dimethyl acetamide; LDA: lithium diisopropylamide; THF: tetrahydrofuran; DCM: dichloromethane; DMF: dimethylformamide; LAH: lithium aluminum hydride.

Because of the limited synthetic methods for 8-aryloxy aporphines and their dependence on non-abundant, naturally occurring aporphine derivatives (*S*-boldine (**1**), (+)-thalicarpine (**43**), and (+)-pakistanamine (**47**)) as starting materials, Ali et al. described an efficient synthesis of 8-phenoxy aporphine **52** as a model substrate for this subset of aporphine dimers by utilizing inexpensive and commercially available starting materials (Scheme 4). The synthesis featured the early stage installation of the required 8-phenoxy group via an aromatic nucleophilic substitution reaction (S_N_Ar), followed by the construction of the aporphine unit using modified Pictet–Spengler cyclization and Pd-XPhos-catalyzed *ortho*-phenol arylation as key steps in this synthesis to generate 8-phenoxy aporphine **52** in a 25% overall yield over six steps. The authors also reported the instability of 8-phenoxy aporphine **52** and its rapid oxidation to 6a,7-dehydroaporphine analog **53**, which raises the possibility that some isolated 8-aryloxy-7-dehydroaporphines may not be naturally occurring and instead be formed through the air oxidation of their parent 8-aryloxy aporphines during various collection, storage, and/or processing operations [50].

## 3. C9-Aryloxy Aporphines and 6a,7-Dehydroaporphines

### 3.1. Chemical Structure Classification and Occurrence

**Figure 4:**
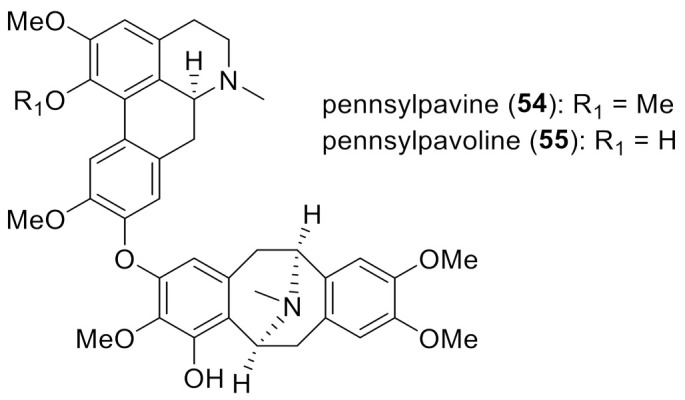
Structures of (−)-pennsylpavine (**54**) and (−)-pennsylpavoline (**55**).

**Figure 5:**
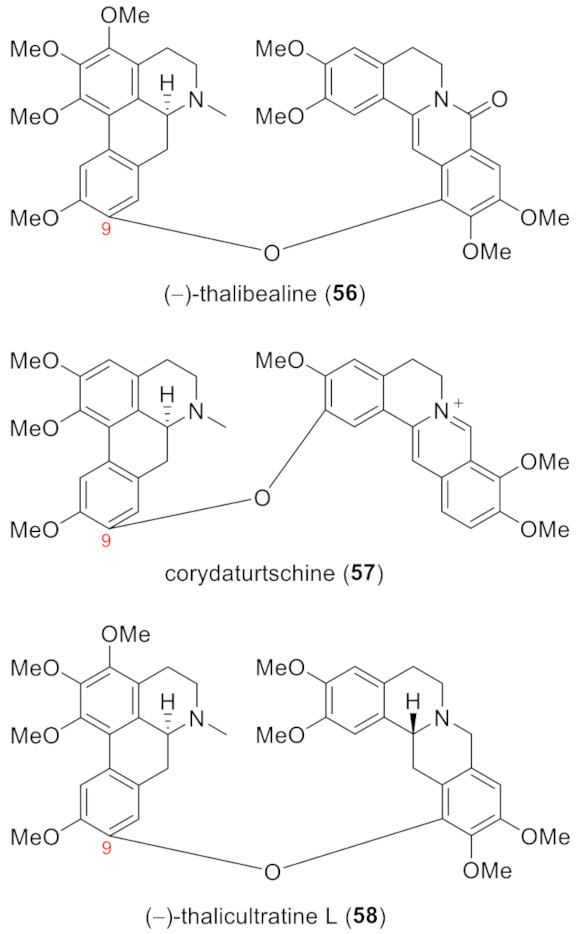
Structures of (−)-thalibealine (**56**), corydaturtschine B (**57**), and (−)-thalicultratine L (**58**).

**Figure 6:**
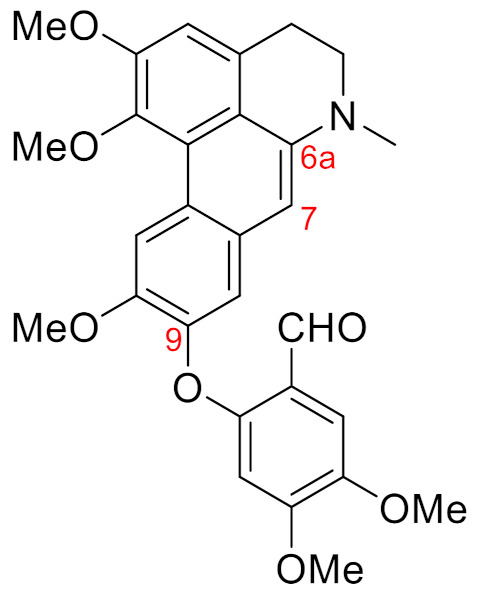
Structure of 6a,7-dehydrohernandaline (**59**).

**Figure 7:**
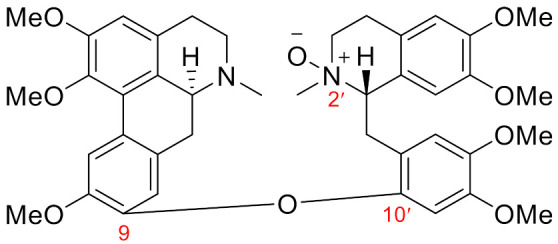
Structure of (+)-thalicarpine-2′-*N*-oxide (**86**).

**Figure 8:**
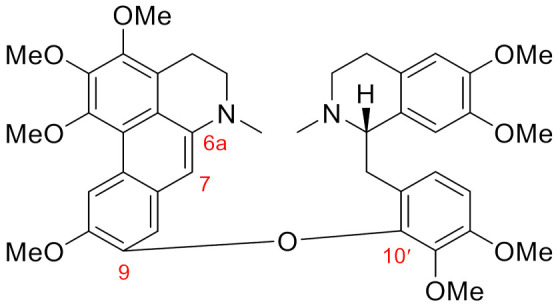
Structure of (+)-6a,7-dehydrohuangshanine (**98**).

**Figure 9:**
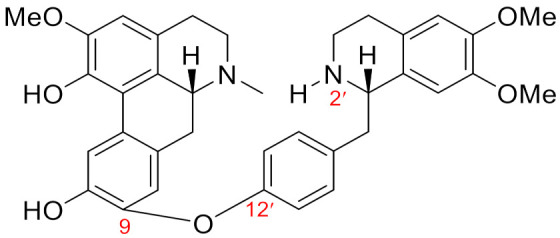
Structure of (+)-2′-norpakistanine (**108**).

**Figure 10:**
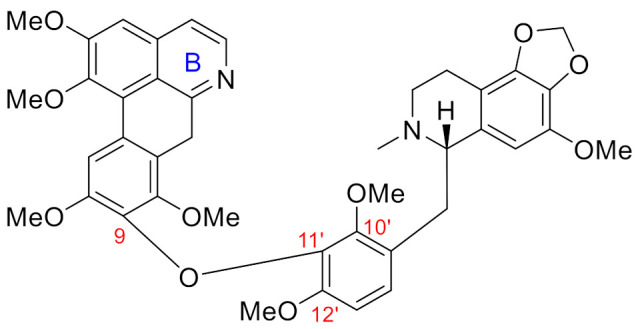
Structure of (+)-przewaline (**115**).

A group of aporphines and 6a,7-dehydroaporphines with an aryloxy group at C9 was isolated from several species of the *Thalictrum* genus (Ranunculaceae), the *Hernandia* genus (Hernandiaceae), the *Berberis* genus (Berberidaceae), *Corydalis turtschaninovii* Bess. (Papaveraceae), and *Tabernaemontana bufalina* Lour. (Apocynaceae). To date, fifty-eight compounds belonging to C9-aryloxy aporphines and five compounds belonging to C9-aryloxy 6a,7-dehydroaporphines have been reported in the literature. This group can be classified according to its chemical structure into four groups:

(1) Aporphine–pavine dimeric alkaloids: in 1974, Shamma and Moniot reported the isolation of (−)-pennsylpavine (**54**) and (−)-pennsylpavoline (**55**) (Figure 4), the only known dimers of this group, from *Thalictrum polygamum* Muhl. [51].
(2) Aporphine–protoberberine dimeric alkaloids: the first member of this group, (−)-thalibealine (**56**) (Figure 5), was isolated in 2001 from the roots of *Thalictrum wangiii* Boivin. [52] and later from the roots of *Thalictrum cultratum* [42]. Additionally, corydaturtschine B (**57**) and (−)-thalicultratine L (**58**) (shown in Figure 5) were isolated from *Corydalis turtschaninovii* Bess. [53] and *Thalictrum cultratum* [42], respectively.
(3) Aporphine and 6a,7-dehydroaporphine–phenyl dimeric alkaloids (hernandaline type): Table 6 shows the names, chemical structures, and botanical sources of seven aporphine–benzyl dimers. Additionally, the structure of 6a,7-dehydrohernandaline (**59**) isolated from *Hernandia sonora* [54] is depicted in Figure 6.
(4) Aporphine and 6a,7-dehydroaporphine–benzylisoquinoline dimeric alkaloids: this is the largest group of C9-aryloxy aporphines and contains fifty reported compounds to date. The names, chemical structures, and botanical sources of these compounds are shown in Table 7, Table 8, Table 9, Table 10, Table 11, Table 12 and Table 13 and Figure 7, Figure 8, Figure 9 and Figure 10. These compounds can be organized, as mentioned in Section 1 (Table 1), according to the location of the ether bond and the substitution pattern on the benzyltetrahydroisoquinoline moiety into four groups; thalicarpine, fetidine, istanbulamine, and pakistanine. Additionally, some compounds do not belong to any of the abovementioned types and constitute a new type of aporphine–benzylisoquinoline dimers linked at C9 of the aporphine unit.

#### 3.1.1. Thalicarpine-Type Dimers

This group, as discussed in Table 1, possesses an ether bond between C9 of the aporphine unit and C10′ of the 12′,13′-disubstituted benzylisoquinoline moiety. Additionally, dimers of this group possess (6a*S*, 1′*S*) absolute configuration. Twenty-four dimers of this type have been found in only two genera: *Thalictrum* (Ranunculaceae) and *Hernandia* (Hernandiaceae). Seventeen aporphine–benzylisoquinolines of the thalicarpine-type dimers **43** and **67**–**82** are listed in Table 7. The first member of this group, (+)-thalicarpine (**43**), was isolated by Kupchan et al. in 1963 [64], and its structure was revised by the same group two years later [65].

**Table 7:**
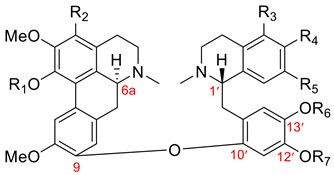
Aporphine–benzylisoquinoline dimeric alkaloids of the thalicarpine type.

Name	R_1_	R_2_	R_3_	R_4_	R_5_	R_6_	R_7_	Botanical Source
(+)-thalicarpine (**43**) (structure revision [65])	Me	H	H	OMe	OMe	Me	Me	*Thalictrum dasycarpum* Fisch. And Lall. [64] *Thalictrum minus* var. *elatum* Jacq. [66] *Thalictrum revolutum* [67] *Hernandia ovigera* L. [65,68,69,70] *Thalictrum minus* L. ssp. elatum [71] *Thalictrum flavum* L. [72] *Thalictrum polygamum* [73] *Thalictrum revolutum* DC [74,75,76] *Thalictrum dioicum* L. [77,78] *Thalictrum minus* L. [79,80] *Thalictrum alpinum* L. [81] *Thalictrum foliolosum* DC [82,83] *Hernandia nymphaeifolia* [56,57]
(+)-thalmelatine (**67**) (structure revision [65])	Me	H	H	OMe	OH	Me	Me	*Thalictrum minus* var. *elatum* Jacq. [66] *Thalictrum minus* L. ssp. elatum [71] *Thalictrum revolutum* DC. [74,75,76] *Thalictrum dioicum * L. [78] *Thalictrum minus* L. [80] *Thalictrum foetidum* L. [84] *Thalictrum minus* L. subsp. *minus* [41]
(+)-adiantifoline (**68**)	Me	OMe	H	OMe	OMe	Me	Me	*Thalictrum minus* L. var. *adiantifolium* Hort. [85,86,87] *Thalictrum minus* L. ssp. elatum [88] *Thalictrum minus* L., race B. [89] *Thalictrum minus* var. *microphyllum* [90] *Thalictrum cultratum* [33] *Thalictrum minus* var. *minus* L. [91] *Thalictrum minus* L. var. *majus* [92] *Thalictrum honanense* W. T. Wang [93]
(+)-O-desmethyladiantifoline (**69**)	Me	OMe	H	OH	OMe	Me	Me	*Thalictrum minus* L. ssp. elatum [88]
(+)-thalmelatidine (**70**) (structure revision [94])	Me	OMe	OCH_2_O		OMe	Me	Me	*Thalictrum minus* L. ssp. elatum [88,95] *Thalictrum minus* var. *microphyllum* [90] *Thalictrum cultratum* [33] *Thalictrum minus* var. *minus* L. [91] *Thalictrum minus* var. *hypoleucum* [96] *Thalictrum minus* L. var. *majus* [92] *Thalictrum honanense* W. T. Wang et S. H. Wang [93]
(+)-thalmineline (**71**)	Me	OMe	OH	OMe	OMe	Me	Me	*Thalictrum minus* L. var. *elatum* Koch. [97,98] *Thalictrum cultratum* [33] *Thalictrum minus* var. *minus* L. [91]
(+)-thalictropine (**72**)	H	H	H	OMe	OMe	Me	Me	*Thalictrum polygamum* [99] *Thalictrum dioicum* L. [78]
(+)-thalictrogamine (**73**)	H	H	H	OMe	OH	Me	Me	*Thalictrum polygamum* [99] *Thalictrum revolutum* DC. [100] *Thalictrum dioicum* L. [78] *Thalictrum foetidum* L. [84]
(+)-thalidoxine (**74**)	Me	H	H	OMe	OMe	Me	H	*Thalictrum dioicum* L. [101]
(+)-pennsylvanine (**75**)	Me	H	H	OMe	OMe	H	Me	*Thalictrum polygamum* Muhl. [102] *Thalictrum revolutum* DC. [75,100] *Thalictrum dioicum* L. [78]
(+)-pennsylvanamine (**76**)	H	H	H	OMe	OMe	H	Me	*Thalictrum polygamum* Muhl. [102] *Thalictrum foetidum* L. [103]
(+)-thalipine (**77**)	Me	H	H	OMe	OH	H	Me	*Thalictrum polygamum* Muhl. [104] *Thalictrum revolutum* DC. [76,100,105] *Thalictrum minus* L. [80] *Thalictrum foetidum* L. [84]
(+)-thalilutidine (**78**)	Me	H	H	OH	OMe	Me	Me	*Thalictrum revolutum* DC. [106]
(+)-thalilutine (**79**)	Me	OH	H	OMe	OMe	Me	Me	*Thalictrum revolutum* DC. [106] *Thalictrum cultratum* [33]
(+)-thaliadanine (**80**)	Me	OMe	H	OMe	OH	Me	Me	*Thalictrum minus* L., race B. [58] *Thalictrum minus* var. *microphyllum* [90]
(+)-bursanine (**81**)	Me	OMe	H	OMe	OH	H	Me	*Thalictrum minus* var. *microphyllum* Boiss. [107]
(+)-methoxyadiantifoline (**82**)	Me	OMe	OMe	OMe	OMe	Me	Me	*Thalictrum omeiense* W.T. Wang [108] *Thalictrum foetidum* L. [109]

Seven more members of the thalicarpine type are shown in Figure 7 and Table 8 and Table 9. Three 2′- or 6-noraporphine–benzylisoquinoline dimers **83**–**85** are listed in Table 8. Additionally, (+)-thalicarpine-2′-*N*-oxide (**86**), the only member of the thalicarpine type with a 2′-*N*-oxide functional group that was isolated from *Hernandia peltate* [110], is depicted in Figure 7. In addition, three 6a,7-dehydroaporphine–benzylisoquinoline dimers **87**–**89** are displayed in Table 9.

**Table 8:**
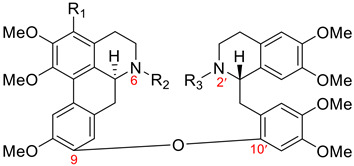
2′-or 6-Noraporphine–benzylisoquinoline dimeric alkaloids of the thalicarpine type.

Name	R_1_	R_2_	R_3_	Botanical Source
(+)-2′-northalicarpine (**83**)	H	Me	H	*Thalictrum revolutum* DC. [111]
(+)-2′-noradiantifoline (**84**)	OMe	Me	H	*Thalictrum minus* L. var. *microphyllum* Boiss. [112]
(+)-6-northalicarpine (**85**)	H	H	Me	*Hernandia peltate* [110]

**Table 9:**
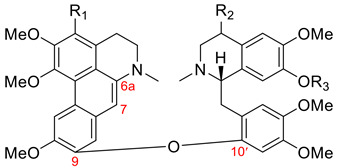
6a,7-Dehydroaporphine–benzylisoquinoline dimeric alkaloids of the thalicarpine type.

Name	R_1_	R_2_	R_3_	Botanical Source
6a,7-dehydrothalicarpine (**87**)	H	H	Me	*Thalictrum minus* L. ssp. elatum [113] *Thalictrum dasycarpum* Fisch. And Lall. [114] *Hernandia ovigera* L. [70]
6a,7-dehydrothalmelatine (**88**)	H	H	H	*Hernandia peltate* [110] *Thalictrum minus* L. subsp. *minus* [41]
(+)-6a,7-dehydromethoxyadiantifoline (**89**)	OMe	OMe	Me	*Thalictrum foetidum* L. [109]

#### 3.1.2. Fetidine-Type Dimers

Nine members of the fetidine type were isolated from the *Thalictrum* genus (Ranunculaceae). Dimers of this group, as described in Table 1, have an ether linkage between C9 of the aporphine unit and C10′ of the 11′,12′-disubstituted benzylisoquinoline moiety. Additionally, they possess (6a*S*, 1′*S*) absolute configuration. Eight aporphine–benzylisoquinolines **90**–**97** of the fetidine type are listed in Table 10. (+)-Fetidine (**90**) is the first representative example of this type that was isolated in 1963 [115], and its structure was revised in 1972 by Cava et al. [116]. Additionally, Figure 8 shows (+)-6a,7-dehydrohuangshanine (**98**), which was found in *Thalictrum faberi* Ulber [31] and represents the only 6a,7-dehydroaporphine–benzylisoquinoline of the fetidine type.

**Table 10:**
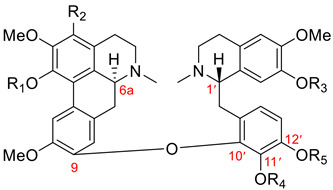
Aporphine–benzylisoquinoline dimeric alkaloids of the fetidine type.

Name	R_1_	R_2_	R_3_	R_4_	R_5_	Botanical Source
(+)-fetidine (**90**) (structure revision [116])	H	H	Me	Me	Me	*Thalictrum foetidum* [115,117,118]
(+)-revolutopine (**91**) (structure revision [25])	Me	H	H	H	Me	*Thalictrum revolutum* DC. [100,105]
(+)-thalirevoline (**92**)	Me	H	Me	Me	H	*Thalictrum revolutum* DC. [100,106]
(+)-thalirevolutine (**93**)	Me	H	Me	Me	Me	*Thalictrum revolutum* DC. [106]
(+)-iznikine (**94**)	Me	OMe	H	H	Me	*Thalictrum minus* var. *microphyllum* Boiss. [107]
(+)-huangshanine (**95**)	Me	OMe	Me	Me	Me	*Thalictrum faberi* Ulber [30]
(+)-faberidine (**96**)	Me	H	H	Me	Me	*Thalictrum faberi* Ulber [31]
(+)-faberonine (**97**)	Me	OMe	H	Me	Me	*Thalictrum faberi* Ulber [31]

#### 3.1.3. Istanbulamine-Type Dimers

As stated in Table 1, dimers of this group have an ether bond between C9 of the aporphine unit and C11’ of the 12’-monosubstituted benzylisoquinoline unit, and they possess (6a*S*, 1′*S*) absolute configuration. The only two members of this group are listed in Table 11.

**Table 11:**
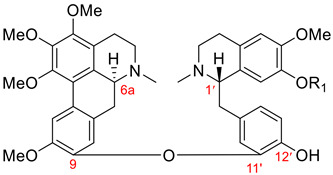
Aporphine–benzylisoquinoline dimeric alkaloids of the fetidine type.

Name	R_1_	Botanical Source
(+)-istanbulamine (**99**)	H	*Thalictrum minus* var. *microphyllum* Boiss. [107]
(+)-thalibulamine (**100**)	Me	*Thalictrum cultratum* [33]

#### 3.1.4. Pakistanine-Type Dimers

Dimers of this group, as described in Table 1, have a characteristic ether linkage between C9 of the aporphine unit and C12′ of the benzylisoquinoline moiety. In contrast to dimers of the thalicarpine, fetidine, and istanbulamine types, these possess (6a*R*, 1′*S*) absolute configuration. Eight aporphine–benzylisoquinoline dimers of this type were isolated from the *Berberis* genus (Berberidaceae). Seven dimers of this type, **101**–**107**, are listed in Table 12, and the structure of (+)-2′-norpakistanine (**108**), isolated from *Berberis valdiviana* Phil. [119], is shown in Figure 9.

**Table 12:**
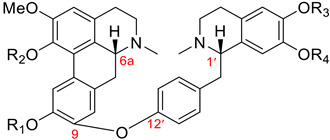
Aporphine–benzylisoquinoline dimeric alkaloids of the pakistanine type.

Name	R_1_	R_2_	R_3_	R_4_	Botanical Source
(+)-pakistanine (**101**)	H	H	Me	Me	*Berberis baluchistanica* Ahrendt [120,121] *Berberis calliobotrys* Bienert ex Aitch. [122] *Berberis orthobotrys* Bienert ex Aitch. [123,124,125] *Berberis empetrifolia* Lam. [62,126,127] *Berberis waziristanica* [128] *Berberis sibirica * [129] *Berberis aristata* [130]
(+)-1-O-methylpakistanine (**102**)	H	Me	Me	Me	*Berberis orthobotrys* Bienert ex Aitch. [124,125] *Berberis calliobotrys* Bienert ex Aitch. [122] *Berberis aristata* [130]
(+)-chitraline (**103**)	H	H	Me	H	*Berberis orthobotrys* Bienert ex Aitch. [124,125] *Berberis zabeliana* Schneider [122] *Berberis calliobotrys* Bienert ex Aitch. [122]
(+)-porveniramine (**104**)	H	H	H	Me	*Berberis empetrifolia* Lam. [131]
(+)-1-O-methylchitraline (**105**)	H	Me	Me	H	*Berberis darwinii* Hook [119]
(+)-waziristanine (**106**)	H	Me	H	Me	*Berberis waziristanica* [128]
1,10-di-O-methylpakistanine (**107**)	Me	Me	Me	Me	*Berberis sibirica* Pall. [132]

#### 3.1.5. New Type of Aporphine–Benzylisoquinoline Dimers

Seven aporphine–benzylisoquinoline dimers, **109**–**115**, that do not fit into any dimeric types listed in Table 1 were isolated in 1998 by Zhang et al. [133]. Their chemical structures possess an ether bond between C9 of the aporphine unit and C11′ of the 10′,12′- or 12′,14′-dimethoxy-substituted benzylisoquinoline moiety. Additionally, they have (6a*S*, 1′*S*) absolute configuration. Six members, **109**–**114**, of this new type are listed in Table 13. Figure 10 depicts the structure of (+)-przewaline (**115**) characterized by an aromatic ring B of the aporphine unit.

**Table 13:**
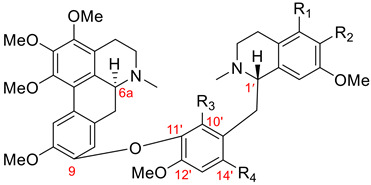
Aporphine–benzylisoquinoline dimeric alkaloids belonging to a new type of dimers.

Name	R_1_	R_2_	R_3_	R_4_	Botanical Source
(+)-przewalskine (**109**)	OCH_2_O		OMe	H	*Thalictrum przewaliskii* [133] *Tabernaemontana bufalina* Lour. [134]
(+)-przewalskinine (**110**)	OCH_2_O		H	OMe	*Thalictrum przewaliskii* [133]
(+)-przewalstine (**111**)	H	OMe	OMe	H	*Thalictrum przewaliskii* [133]
(+)-przewalstinine (**112**)	H	OMe	H	OMe	*Thalictrum przewaliskii* [133]
(+)-przewalstidine (**113**)	OH	OMe	OMe	H	*Thalictrum przewaliskii* [133]
(+)-przewalstidinine (**114**)	OH	OMe	H	OMe	*Thalictrum przewaliskii* [133]

### 3.2. Biological Activities of C9-Aryloxy Aporphines and 6a,7-Dehydroaporphines

Though most of the C8-aryloxy aporphines have shown significant cytotoxicity activities, only a few members of the C9-aryloxy aporphines have been reported to exhibit cytotoxic effects. Among them, thalicarpine is the most important member that entered into clinical trials.

In 1963, Kupchan et al. noted the transient hypotensive effects of thalicarpine (**43**) in cats with doses ranging from 0.5 to 5 mg/kg [135]. In subsequent studies with thalicarpine (**43**), Kupchan et al. reported that it had significant antitumor activity against Walker 256 carcinosarcoma in rats over a wide range of dosages [136,137,138]. Moreover, the in vitro cytotoxicities of some alkaloids from *Thalictrum minus* on a monolayer cell culture of KB cells were determined by measuring the degree of protein synthesis inhibition. Hernandaline (**60**), thalicarpine (**43**), and thalmelatine (**67**) showed 100% protein synthesis inhibition at a concentration of 100 μg/mL. However, dehydrothalicarpine (**87**) was devoid of activity [139]. In another study, thalicarpine (**43**) showed strong inhibitory activity against the HeLa-S3 cell line, with ED_50_ = 2.5 μg/mL [140]. To understand the mode of action responsible for the antitumor activity of thalicarpine (**43**), Allen and Creaven showed that it has significant effects on macromolecular biosynthesis in cultured L1210 mouse leukemia cells at a concentration of 100 μM. It suppressed DNA, RNA, and protein synthesis by inhibiting the incorporation of thymidine, uridine, and leucine into DNA, RNA, and protein, respectively. Additionally, the authors stated that they could not detect any significant binding of thalicarpine (**43**) to calf thymus DNA using ultraviolet spectroscopy [137]. In a subsequent study using equilibrium dialysis, the same authors reported that tritium-labeled thalicarpine (**43**) reversibly bound to calf thymus DNA in vitro. They also found that thalicarpine (**43**) did not bind to human serum albumin in vitro, but it did bind to an unidentified human serum component in vivo [141]. The in vitro inhibition of DNA, RNA, and protein synthesis by thalicarpine (**43**) and its binding to DNA was also reported in a later study by Creasey. The authors also found that treatment of mice bearing an S180 tumor with thalicarpine at doses of 30 and 60 mg/kg led to the 48 and 83% inhibition, respectively, of thymidine incorporation into DNA after 24 h [142]. Moreover, Allen and Creaven found that at relatively high concentrations of 0.1–0.5 mM, thalicarpine (**43**) inhibited rat liver microsomal aniline hydroxylase activity in vitro [143]. Based on its significant cytotoxicity activity in vitro, thalicarpine (**43**) (NSC 68075) was selected by National Cancer Institute (NCI) for clinical evaluation. It underwent numerous preclinical toxicological and pharmacological studies on mice, rats, cats, hamsters, dogs, and monkeys [144,145,146,147,148,149,150,151,152,153,154,155] before proceeding into initial clinical trials. Phase I clinical trials with 34 patients at a dose of 1400 mg/m^2^ did not show promising antitumor activity, and the patients encountered adverse reactions of the central nervous and/or cardiovascular systems [156]. Allen et al. examined the plasma decay and urinary excretion of tritiated thalicarpine (**43**) in 19 patients at doses of 300–1900 mg/m^2^. They found that urinary excretion was very slow, with only 19.84% of the dose being excreted over 12 days in nine patients. The data indicated the prolonged body retention of thalicarpine (**43**), with significant localization in tissues. This raised the probability of dangerous drug accumulation if it is given at short dosage intervals [157]. Because of the absence of any hematologic, hepatic, and renal toxicity of thalicarpine during the phase I clinical trial, it entered into phase II trial with a recommended dose of 1100 mg/m^2^. Unfortunately, the results from 14 patients did not support further clinical investigation [158].

Though clinical trials of thalicarpine (**43**) were terminated, many research groups continued cytotoxic studies on this compound. For example, Todorov et al. found that thalicarpine (**43**) entrapped in negatively charged liposome carriers exhibited antitumor activity against Walker S and lymphoma TLX5 cell lines 2.5 times more potently than free thalicarpine (**43**) [159,160,161]. In 1980, Stoychkov et al. showed that thalicarpine (**43**) enhanced the antitumor activity of cyclophosphamide, a known antitumor drug, against sarcoma 37, Lewis lung carcinoma (LLC), and L1210 leukemia in mice. This synergetic effect was attributed to the different pharmacological behavior and mechanisms of action of the two drugs [162,163]. In a subsequent study, the combination therapy of cyclophosphamide with different cytotoxic drugs such as thalicarpine (**43**), carmustine (BCNU), lomustine (CCNU), 5-fluorouracil, and methotrexate in mice with lymphoid leukemia L-1210 showed that normally ineffective doses of cyclophosamide were active when combined only with thalicarpine (**43**). The other drugs exhibited either no synergetic effect or even decreased the antitumor activity of cyclophosphamide [164]. In 1992, the in vitro antitumor activity of thalicarpine (**43**) against cisplatin-sensitive and cisplatin-resistant rat ovarian tumor cell lines was examined, and the ID_50_ values were found to be 39.3 and 27.3 µg/mL, respectively. Furthermore, combination therapy using cisplatin and thalicarpine (**43**) displayed an additive antitumor effect in cisplatin-sensitive cells but not cisplatin-resistant cells. The authors suggested thalicarpine (**43**) as a possible candidate for the treatment of cisplatin-resistant ovarian tumors [165].

One of the major obstacles in cancer treatment is the development of drug resistance and even multidrug resistance (MDR). A commonly used technique to suppress the development of drug resistance is combination therapy in which two or more drugs acting by different modes of action and/or at different biomolecular targets can be used together. In a study by Gupta et al., sixty-two anti-cancer drugs were tested against puromycin resistant (Pur^R^) mutants of HeLa cells to determine their ability to develop cross-resistance in this cell line. Thalicarpine (**43**) was one of the drugs that showed no cross-resistance and was suggested as a drug that can be used in combination therapy to overcome MDR [166]. Subsequently, the ability of thalicarpine (**43**) to reverse MDR in Adriamycin (ADR)-resistant murine leukemic P388/R-84 cells was investigated by Krishan et al. They found that: (1) 2 µM of thalicarpine (**43**) reduced the ID_50_ of Adriamycin from 10.8 to 1.4 µM after 1 h of exposure and from 3.5 to 0.07 µM after 24 h of continuous exposure to the drug combination, (2) flow cytometric analysis showed that Adriamycin retention in P388/R-84 cells was enhanced by thalicarpine (**43**) in a time- and dose-dependent fashion, (3) thalicarpine (**43**) partially inhibited the binding of tritiated azidopine with P-glycoprotein. These results indicated that thalicarpine’s ability to overcome MDR via the enhancement of ADR cellular retention may be attributed to its interaction with P-glycoprotein efflux binding sites [167]. Numerous other studies on the mechanism of action of thalicarpine (**43**) reversing MDR in a variety of resistant cell lines such as cisplatin-resistant rat ovarian tumor cells [168,169,170], an Adriamycin-resistant human breast cancer cell line (MCF/AdR) [171,172], multidrug-resistant human colon carcinoma cells [173], multidrug-resistant mouse lymphoma cells [174], and doxorubicin (DOX)-resistant HL-60 human acute myeloid leukemia (HL-60/DOX) [175]—as well as in artificial membranes composed of neutral and negatively charged phospholipids [176,177]—have been reported.

Lin et al. reported that huangshanine (**95**) showed cytotoxicity against Walker 256 carcinoma cells [30]. In 1996, Chen’s group reported the in vitro cytotoxic activities of thalicarpine (**43**) and hernandaline (**60**) against P-388, KBI6, A-549, and HT-29 cell lines, with ED_50_ values ranging from 1.9 to 5.4 μg/mL for thalicarpine (**43**) and from 1.8 to 3.1 μg/mL for hernandaline (**60**) [56]. 

In addition to cytotoxicity activity, some C9-aryloxy dimeric aporphines have shown in vitro antimicrobial activity against *Mycobacterium smegmatis* at a minimum inhibitory concentration of 100 µg/mL. These alkaloids are thalicarpine (**43**) [73,75], thalipine (**77**) [100], thalirevoline (**92**) [106], thaliadanine (**80**) [58], thalmelatine (**67**), and pennsylvanine (**75**) [75].

In 1988, the effect of methoxyadiantifoline (**82**) on the cardiovascular system was studied. It was found to increase the coronary blood flow in guinea pig hearts by 12.9% and 20.4% at 17 and 50 μM, respectively, and this action was accompanied by negative chronotropic and inotropic effects [178]. In another report, Jie et al. studied the effect of methoxyadiantifoline (**82**) on the physiological properties of the myocardium in isolated left and right rat atria. They found that at 30 μM, the functional refractory period of left atria muscles was prolonged from 61 to 90 ms after 15 min and contractility decreased to 50% after 40 min. Additionally, the rate and contractility of rat right atria were decreased to 60% and 50%, respectively [108]. In 1991, the in vitro antifibrotic activity of methoxyadiantifoline (**82**) was evaluated. The results showed that the compound displayed a significant binding affinity for membrane lipids and alveolar macrophages that resulted in its potent inhibition of zymosan-stimulated oxygen consumption (100% inhibition at 79 μM), superoxide release (85% inhibition at 79 μM), and hydrogen peroxide secretion (100% inhibition at 33 μM) by alveolar macrophages. The authors stated the potential effectiveness of methoxyadiantifoline (**82**) as an antifibrotic drug [179].

Chen et al. determined the in vitro anti-platelet aggregation effects of constituents from *Hernandia nymphaeifolia*. The results demonstrated that thalicarpine (**43**) completely inhibited both collagen (10 µg/mL) and platelet-activating factor (PAF; 2 nM)-induced platelet aggregation at 50 µg/mL. At the same concentration, hernandaline (**60**), showed the moderate inhibition of platelet aggregation induced by arachidonic acid (AA; 100 µM) and collagen (10 µg/mL) and the nearly complete inhibition of platelet aggregation induced by PAF. On the other hand, dehydrohernandaline (**59**) exhibited weak anti-platelet aggregation effects induced by AA, collagen, and PAF [57]. In another anti-platelet aggregation study, thalicarpine (**43**), at 100 µM, exhibited the nearly complete inhibition of platelet aggregation induced by collagen and the moderate inhibition of platelet aggregation induced by both AA and PAF [180].

In 2001, the in vitro vasorelaxant properties of constituents from *Hernandia nymphaeifolia* were studied by Chen et al. They found that hernandaline (**60**) (at 100 μM) completely inhibited aortic contraction induced by norepinephrine (3 μM) and only inhibited 27% of the aortic contraction induced by high K^+^ (80 mM). However, 6a,7-dehydrohernandaline (**59**) exhibited no inhibitory activity due to the aromatization of ring C [181].

Recently, the immunosuppressive activity of twenty-one different isoquinoline alkaloids was tested in vitro on the concanavalin A (Con A)-stimulated proliferation of mice splenocytes. The aporphine–benzyl dimers (+)-thaliadine (**61**), (−)-6a*R*-2′-methoxycarbonyl-thaliadine (**64**), (−)-6a*R*-2′-carboxyl-thaliadine (**65**), and (−)-6a*R*-3-methoxy-hernandalinol (**66**) were among the tested compounds, and only **65** showed significant a inhibition of T lymphocytes, with IC_50_ = 43.9 μM [63].

### 3.3. Reported Synthetic Approaches for C9-Aryloxy Aporphines

**Scheme 5:**
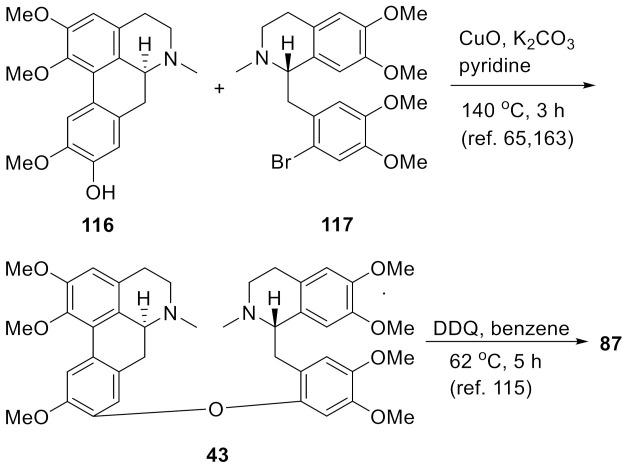
Thalicarpine (**43**) and 6a,7-dehydrothalicarpine (**87**) syntheses via the late-stage installation of the ether bond using a classical Ullman coupling reaction followed by DDQ (2,3-dichloro-5,6-dicyano-l,4-benzoquinone) oxidation.

Few synthetic approaches for 9-aryloxy aporphines of the hernandaline or thalicarpine-type dimers have been reported in the literature. The earliest strategy, shown in Scheme 5, for the synthesis of the potent antitumor agent thalicarpine (**43**) was described by Kupchan and coworkers in 1965. A late-stage ether linkage formation using an Ullman coupling of naturally occurring aporphine (*S*)-*N*-methyllaurotetanine (**116**) and (*S*)-6′-bromolaudanosine (**117**) (synthesized from (*S*)-laudanosine [182]) gave thalicarpine (**43**) in a low yield [65,183]. Following the same approach, the naturally occurring hernandaline (**60**) [56] and adiantifoline (**68**) [184,185] were synthesized in 79% and 21% yields, respectively. Additionally, 6a,7-dehydrothalicarpine (**87**) was synthesized in a 47% yield via the oxidation of thalicarpine (**43**) using 2,3-dichloro-5,6-dicyano-l,4-benzoquinone (DDQ) in benzene (Scheme 5) [114]. 

**Scheme 6:**
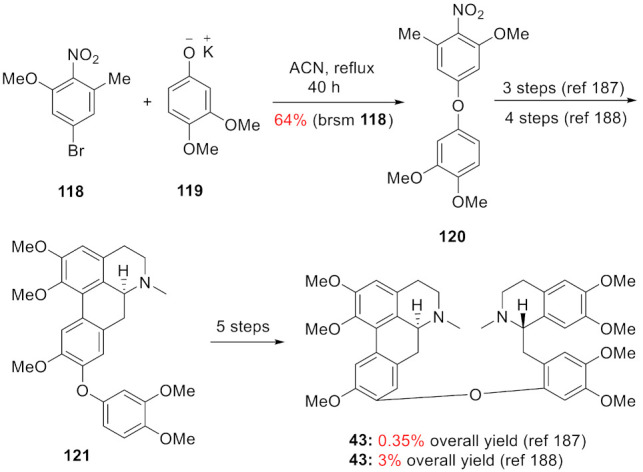
Thalicarpine (**43**) synthesis via the early-stage installation of the diaryl ether linkage. brsm: based on recovered starting material.

Because of the limited accessibility of those naturally occurring starting materials and the low yield of Ullman coupling reactions, Kupchan et al. presented an alternative strategy for thalicarpine (**43**) synthesis via the installation of the biaryl ether bond at an early stage of the synthesis. As illustrated in Scheme 6, diphenyl ether **120** was synthesized via an S_N_Ar reaction followed by the elaboration of the aporphine and benzyltetrahydroisoquinoline units to afford thalicarpine (**43**), though in only a 0.35% overall yield [186]. Some modifications in the formation of the aporphine unit from intermediate **120** enhanced the overall yield of thalicarpine (**43**) to 3% using the same strategy [187]. 

**Scheme 7:**
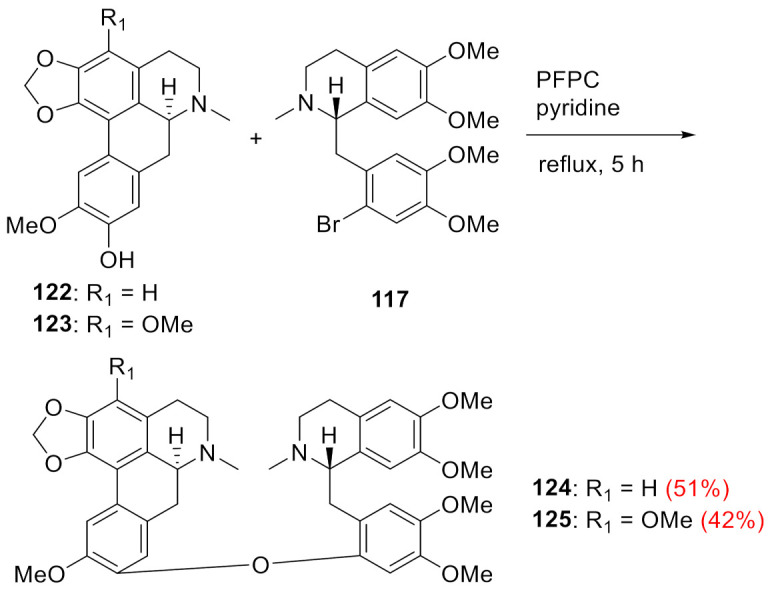
Synthesis of unnatural dimers **124** and **125** using an improved Ullman coupling. PFPC: pentafluorophenylcopper.

Despite the low yield of the Ullman coupling reaction, the late-stage installation of ether linkage has the advantage that the two units of the dimer may be separately synthesized as pure enantiomers, which allows for the structural and stereochemical variation of the two halves before the final coupling step. Due to this advantage, Cava et al. described an improved Ullmann diphenyl ether synthesis using pentafluorophenylcopper (PFPC) as the coupling agent, and its application in the syntheses of non-natural aporphine–benzylisoquinoline dimers **124** and **125** in 51% and 42% yields, respectively (Scheme 7) [188].

**Scheme 8:**
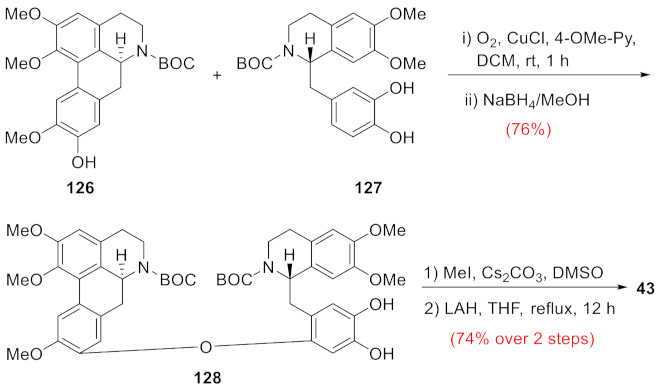
Thalicarpine (**43**) synthesis via catalytic aerobic cross-dehydrogenative coupling. Py: pyridine; DCM: dichloromethane; DMSO: dimethylsulfoxide; LAH: lithium aluminum hydride; THF: tetrahydrofuran.

In 2019, Jean-Philip Lumb and coworkers reported a catalytic aerobic cross-dehydrogenative coupling reaction of phenols and catechols, and its application in thalicarpine (**43**) synthesis in 20% overall yield over 11 steps (longest linear sequence). As depicted in Scheme 8, the coupling of phenol **126** and catechol **127** using CuCl under an atmosphere of O_2_ generated intermediate **128** in a 76% yield, which, after methylation and reduction, afforded thalicarpine (**43**) in a 74% yield over two steps [189]. 

**Scheme 9:**
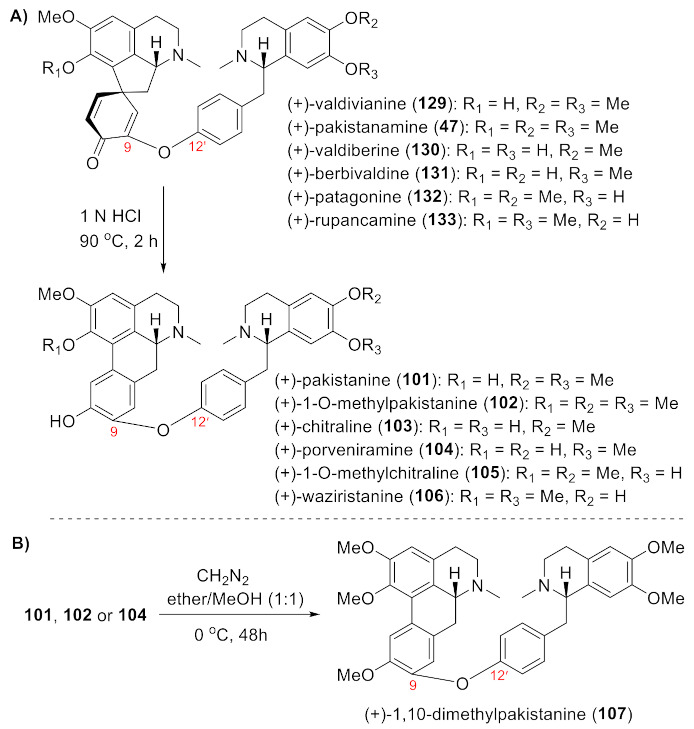
(**A**) Synthesis of pakistanine-type dimers via the acid-catalyzed rearrangement of pakistanamine-type dimers. (**B**) Synthesis of (+)-1,10-dimethylpakistanine (**107**) via the methylation of pakistanine-type dimer derivatives.

On the other hand, the syntheses of naturally-occurring C9-aryloxy aporphines of the pakistanine-type dimers **101**–**107** were mainly achieved through the acid-catalyzed rearrangement of the naturally occurring proaporphine–benzylisoquinoline dimers of the pakistanamine-type dimers. As shown in Scheme 9A, heating proaporphine–benzylisoquinoline dimers **129**, **47**, and **130**–**133** in dilute hydrochloric acid furnished pakistanine-type dimers **101**–**106**, respectively. However, the yields for these transformations were not reported [121,131,190]. Additionally, (+)-1,10-dimethylpakistanine (**107**) was synthesized in a 76–78% yield via the methylation of pakistanine-type dimers **101**, **102**, or **104** using an ethereal diazomethane solution [121,131].

## 4. C11-Aryloxy Aporphines (Kalashine Type)

### 4.1. Chemical Structure Classification and Occurrence

Only two 11-aryloxy aporphines (Table 14) of the kalashine-type dimers were isolated from the *Berberis* genus (Berberidaceae) to date. As stated in Table 1, kalashine-type dimers have an ether bond between C11 of the aporphine and C12′ of the benzylisoquinoline unit, and they possess (6a*R*, 1′*S*) absolute configuration. There are no reported pharmacological activities for 11-aryloxy aporphines.

### 4.2. Reported Synthetic Approaches for C11-Aryloxy Aporphines

**Scheme 10:**
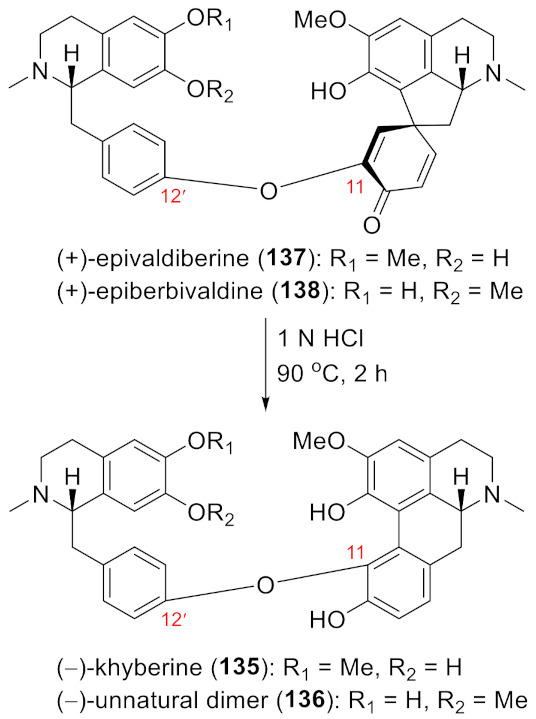
Synthesis of kalashine-type dimers via the acid-catalyzed rearrangement of epivaldiberine-type dimers.

As depicted in Scheme 10, (−)-khyberine (**135**) and the non-natural dimer **136** were prepared via the acid-catalyzed rearrangement of the naturally occurring proaporphine–benzylisoquinoline dimers **137** and **138** of the epivaldiberine type [131,190].

## 5. Summary

In this review, we reported the nomenclature, chemical structures, biological activities, and synthetic approaches of dimeric aporphines with an aryloxy substituent at C8, C9, or C11 of the aporphine unit. To the best of our knowledge, a total of ninety-seven dimers have been isolated from the *Thalictrum* genus (Ranunculaceae), *Dehaasia triandra* (Lauraceae), the *Hernandia* genus (Hernandiaceae), the *Berberis* genus (Berberidaceae), *Corydalis turtschaninovii* Bess. (Papaveraceae), and *Tabernaemontana bufalina* Lour. (Apocynaceae). The most represented class are C9-aryloxy aporphines and 6a,7-dehydroaporphines, while the C11-aryloxy aporphines are the rarest. Several members of these dimeric aporphines have exhibited cytotoxic activities. Only (+)-thalicarpine (**43**) entered clinical trials, which were terminated due to a lack of sufficient antitumor activity. In addition, some of these dimeric aporphines have shown antimalarial, antibacterial, antifibrotic, anti-platelet aggregation, vasorelaxant, and immunosuppressive activities, thus demonstrating the variety of pharmacology types that can be explored with this set of molecular scaffolds. Several synthetic approaches have been reported for the preparation of these natural products, such as the enzymatic catalyzed oxidation of aporphine and the photo- and acid-catalyzed rearrangement of naturally occurring proaporphine–benzylisoquinoline dimers. In addition, three approaches have been reported for the diaryl ether bond formation: (1) late-stage installation via the Ullman coupling of phenols and aryl halides; (2) late-stage installation through the catalytic aerobic cross-dehydrogenative coupling of phenols and catechols; and (3) early-stage installation using aromatic nucleophilic substitution reactions. However, additional opportunities for synthetic method development to access this class of natural products and the potential for generating non-natural derivatives remain.

## Figures and Tables

**Table 1:**
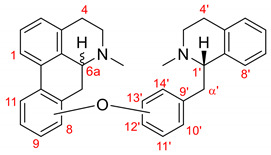
Location of the ether bond in different types of aporphine–benzylisoquinoline dimeric alkaloids.

Dimeric Types	Location of the Ether Bond	6a
Thalicarpine type	C9 of the aporphine unit and C10’ of the disubstituted 12′,13′-benzylisoquinoline moiety	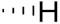
Fetidine type	C9 of the aporphine unit and C10’ of the disubstituted 11′,12′-benzylisoquinoline moiety	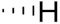
Istanbulamine type	C9 of the aporphine unit and C11’ of the monosubstituted 12′-benzylisoquinoline moiety	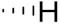
Thalifaberine type	C8 of the aporphine unit and C12’ of the benzylisoquinoline moiety	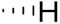
Pakistanine type	C9 of the aporphine unit and C12’ of the benzylisoquinoline moiety	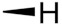
Kalashine type	C11 of the aporphine unit and C12’ of the benzylisoquinoline moiety	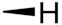

**Table 2:**
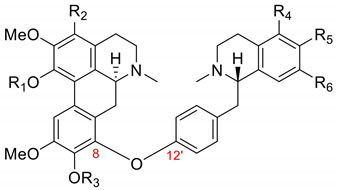
Aporphine–benzylisoquinoline dimeric alkaloids of the thalifaberine type.

Name	R_1_	R_2_	R_3_	R_4_	R_5_	R_6_	Botanical Source
(+)-thalifaberine (**8**)	Me	OMe	Me	H	OMe	OMe	*Thalictrum faberi* Ulber [30,31] *Thalictrum cultratum* [32,42] *Thalictrum atriplex* [40]
(+)-thalifabine (**9**)	Me	OMe	Me	OMe	–OCH_2_O–		*Thalictrum faberi* Ulber [30,31]
(+)-thalifarapine (**10**) (also known as (+)-thalifaroline)	Me	OH	Me	H	OMe	OMe	*Thalictrum faberi* Ulber [31] *Thalictrum cultratum* [33,43]
(+)-thalifabatine (**11**)	Me	OMe	Me	OH	OMe	OMe	*Thalictrum faberi* Ulber [31] *Thalictrum cultratum* [42]
(+)-thalifasine (**12**)	Me	OH	Me	OH	OMe	OMe	*Thalictrum faberi* Ulber [31]
(+)-thalifaronine (**13**)	Me	H	Me	H	OMe	OMe	*Thalictrum cultratum* [32,33,42] *Thalictrum faberi* Ulber [38]
(+)-thalifaramine (**14**)	Me	H	Me	H	OMe	OH	*Thalictrum cultratum* [32,33] *Thalictrum faberi* Ulber [38]
(+)-thalifaretine (**15**)	Me	OMe	Me	H	OMe	OH	*Thalictrum cultratum* [32,33] *Thalictrum artiplex* [40]
(+)-thalifaricine (**16**)	Me	OMe	H	H	OMe	OH	*Thalictrum cultratum* [32,33] *Thalictrum faberi* Ulber [38]
(+)-thalifarazine (**17**)	Me	OMe	Me	H	OH	OMe	*Thalictrum cultratum* [32,33] *Thalictrum sessile* Hayata [36,37] *Thalictrum faberi* Ulber [38]
(+)-thalifalandine (**18**)	H	OMe	Me	H	OMe	OMe	*Thalictrum faberi* Ulber [34]
(+)-thalifaboramine (**19**)	Me	H	H	H	OMe	OMe	*Thalictrum faberi* Ulber [35] *Thalictrum minus* L. *subsp. minus* [41]
(+)-thalifaberidine (**20**)	Me	OMe	H	H	OH	OMe	*Thalictrum faberi* Ulber [38]
(+)-3-hydroxy-6′-desmethyl-9-O-methylthalifaboramine (**21**)	Me	OH	Me	H	OH	OMe	*Thalictrum faberi* Ulber [39] *Thalictrum minus* L. *subsp. minus* [41]
(+)-3-hydroxymethylthalifaboramine (**22**)	Me	OH	H	H	OMe	OMe	*Thalictrum faberi* Ulber [39]
(+)-6′-des methylthalifaboramine (**23**)	Me	H	H	H	OH	OMe	*Thalictrum faberi* Ulber [39]
(+)-3,5′-dihydroxy thalifaboramine (**24**)	Me	OH	H	OH	OMe	OMe	*Thalictrum faberi* Ulber [39]
(+)-5′-dihydroxy thalifaboramine (**25**)	Me	H	H	OH	OMe	OMe	*Thalictrum faberi* Ulber [39]
(+)-3-hydroxy-6′-des methylthalifaboramine (**26**)	Me	OH	H	H	OH	OMe	*Thalictrum faberi* Ulber [39]
(+)-thalicultratine A (**27**)	Me	H	Me	OH	OMe	OMe	*Thalictrum cultratum* [42]
(+)-thalicultratine B (**28**)	Me	OMe	Me	OMe	OMe	OMe	*Thalictrum cultratum* [42]
(+)-thalicultratine C (**29**)	Me	OMe	Me	–OCH_2_O–		OMe	*Thalictrum cultratum* [42]

**Table 3:**
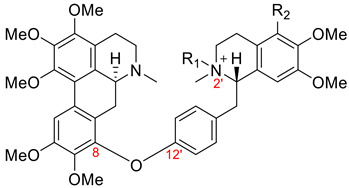
2′-*N*-oxide aporphine–benzylisoquinoline dimeric alkaloids of the thalifaberine type.

Name	R_1_	R_2_	Botanical Source
(+)-thalicultratine H (**30**)	αO	OMe	*Thalictrum cultratum* [42]
(+)-thalicultratine I (**31**)	βO	OMe	*Thalictrum cultratum* [42]
(+)-thalicultratine J (**32**)	αO	OH	*Thalictrum cultratum* [42]
(+)-thalicultratine K (**33**)	βO	OH	*Thalictrum cultratum* [42]

**Table 4:**
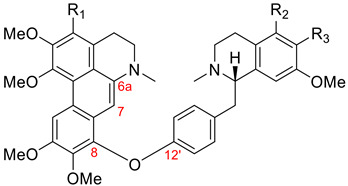
6a,7-Dehydroaporphine–benzylisoquinoline dimeric alkaloids of the thalifaberine type.

Name	R_1_	R_2_	R_3_	Botanical Source
(+)-thalicultratine D (**34**)	H	H	OMe	*Thalictrum cultratum* [42]
(+)-thalicultratine E (**35**)	OMe	OH	OMe	*Thalictrum cultratum* [42]
(+)-thalicultratine F (**36**)	OMe	OMe	OMe	*Thalictrum cultratum* [42]
(+)-thalicultratine G (**37**)	OMe	–OCH_2_O–		*Thalictrum cultratum* [42]
(+)-dehydrothalifaberine (**38**)	OMe	H	OMe	*Thalictrum faberi* Ulber [31] *Thalictrum artiplex* [40] *Thalictrum cultratum* [42]

**Table 5:**
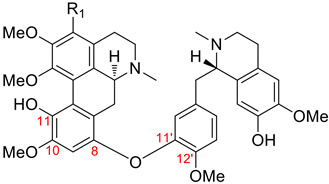
Aporphine–benzylisoquinoline dimeric alkaloids belonging to a new type of reticuline–reticuline dimers.

Name	R_1_	Botanical Source
(+)-faurithaline (**39**)	H	*Thalictrum fauriei* [43]
(+)-3-methoxyfaurithaline (**40**)	OMe	*Thalictrum fauriei* [43]

**Table 6:**
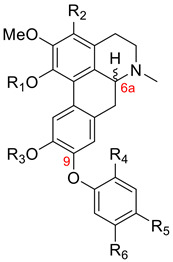
Aporphine–phenyl dimeric alkaloids (hernandaline type).

Name	6a	R_1_	R_2_	R_3_	R_4_	R_5_	R_6_	Botanical Source
(+)-hernandaline (**60**)	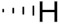	Me	H	Me	CHO	OMe	OMe	*Hernandia ovigera* L. [55] *Hernandia nymphaeifolia* [56,57]
(+)-thaliadine (**61**)	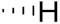	Me	OMe	Me	CHO	OMe	OMe	*Thalictrum minus* race B [58] *Thalictrum minus* L. ssp. Majus [59] *Thalictrum elegans* [60]
(−)-natalinine (**62**)	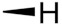	H	H	H	H	CHO	H	*Berberis empetrifolia* [61]
(−)-natalamine (**63**)	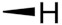	H	H	H	H	CH_2_OH	H	*Berberis empetrifolia* Lam. [62]
(−)-6a*R*-2′-methoxy carbonyl-thaliadine (**64**)	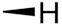	Me	OMe	Me	CO_2_Me	OMe	OMe	*Thalictrum cirrhosum* Levl. [63]
(−)-6a*R*-2′-carboxyl -thaliadine (**65**)	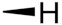	Me	OMe	Me	COOH	OMe	OMe	*Thalictrum cirrhosum* Levl. [63]
(−)-6a*R*-3-methoxy- hernandalinol (**66**)	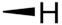	Me	OMe	Me	CH_2_OH	OMe	OMe	*Thalictrum cirrhosum* Levl. [63]

**Table 14:**
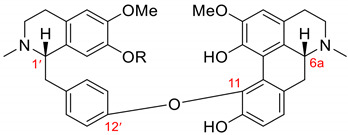
Aporphine–benzylisoquinoline dimeric alkaloids of the kalashine type.

Name	R	Botanical Source
(−)-kalashine (**134**)	Me	*Berberis orthobotrys* Bienert ex Aitch [123,124] *Berberis calliobotrys* Bienert ex Aitch [122]
(−)-khyberine (**135**)	H	*Berberis calliobotrys* Bienert ex Aitch [122]

## Data Availability

Not applicable.
